# Nature against Diabetic Retinopathy: A Review on Antiangiogenic, Antioxidant, and Anti-Inflammatory Phytochemicals

**DOI:** 10.1155/2022/4708527

**Published:** 2022-03-09

**Authors:** Mohammad Amin Khazeei Tabari, Razie Mirjalili, Hooman Khoshhal, Elahe Shokouh, Mohanna Khandan, Elham Hasheminasabgorji, Ali Hafezi-Moghadam, Abouzar Bagheri

**Affiliations:** ^1^Student Research Committee, Mazandaran University of Medical Sciences, Sari, Iran; ^2^USERN Office, Mazandaran University of Medical Sciences, Sari, Iran; ^3^Department of Clinical Biochemistry and Medical Genetics, Faculty of Medicine, Immunogenetics Research Center, Mazandaran University of Medical Sciences, Sari, Iran; ^4^Molecular Biomarkers Nano-Imaging Laboratory, Brigham and Women's Hospital, Boston, MA, USA; ^5^Department of Radiology, Harvard Medical School, Boston, MA, USA

## Abstract

*Background and Purpose*. Diabetes mellitus (DM), hyperglycemia, and hypertension can result in diabetic retinopathy (DR), which is a major cause of blindness on a global scale. Development of DR is associated with decreased endothelial cells, increased basal membrane thickness, permeation of the retinal blood barrier, and neovascularization in patients. The purpose of the present review is to provide an overview of the findings regarding applications of phytochemicals for DR treatment and could be a beneficial resource for further clinical studies and also a basis for pharmaceutical purposes for drug design. *Materials and Methods*. A narrative literature review was performed from electronic databases including Web of Science, PubMed, and Scopus to analyze the effects of different phytochemicals to prevent or treat oxidation, angiogenesis, and inflammation in diabetic retinopathy. The inclusion criteria were original studies, which included the effects of different phytochemicals on diabetic retinopathy. The exclusion criteria included studies other than original articles, studies which assessed the effects of phytochemicals on nondiabetic retinopathy, and studies which used phytochemical-rich extracts. *Results and Conclusions*. Studies have shown that increased levels of inflammatory cytokines, angiogenic, and oxidative stress factors are involved in the progression and pathogenesis of DR. Therefore, phytochemicals with their anti-inflammatory, antiangiogenic, and antioxidant properties can prevent DR progression and retinal damage through various cellular mechanisms. It is also shown that some phytochemicals can simultaneously affect the inflammation, oxidation, and angiogenesis in DR.

## 1. Introduction

Diabetic retinopathy (DR) is one of the important microvascular complications of diabetes mellitus (DM), in which chronic hyperglycemia and hypertension affect the retinal microvasculature and cause critical damage leading to blindness worldwide [1–4]. It is estimated that, by 2030, the number of people with diabetes will exceed 500 million, making it a global threat, and one-third of people will have DR [5, 6]. Decreased endothelial cells, increased basal membrane thickness, permeation of the retinal blood barrier, and neovascularization are some of the impairments seen in patients with DR [7]. DR has two stages based on the severity of the symptoms: nonproliferative DR (NPDR) and proliferative DR (PDR). NPDR is associated with microaneurysm and “dot and blot” hemorrhages, whereas PDR is characterized by retinal neovascularization. PDR is also associated with retinal hemorrhage and detachment, resulting in partial or complete loss of vision. Loss of central vision occurs due to increased retinal vessel permeability and edema [8]. Retinal neovascularization plays an important role in the pathogenesis of DR [1]. Vascular endothelial growth factor (VEGF) is an angiogenic factor that is expressed in a large number of retinal cells exposed to hyperglycemia. This factor increases proliferation, migration, and tubal formation. Therefore, in patients with DR, VEGF levels in the retina significantly increase, which is directly related to the progression of the disease [1, 9]. VEGF antagonists can be used as a treatment to reduce angiogenesis in patients with DR. Inflammation and oxidative stress are important in the pathogenesis and development of DR [10, 11]. Chronic hyperglycemia in patients with DR causes high production of inflammatory cytokines, including tumor necrosis factor (TNF-*α*) and interleukin (IL-1*β*), and subsequently destroys retinal cells [12]. In the inflammatory process of patients with DR, hyperglycemia increases the expression of nuclear factor kappa B (NF-*κ*B), which causes many disorders, including increased retinal vascular leakage, leukocytosis, increased expression of inflammatory cytokines, and intercellular adhesion molecule-1 (ICAM-1) [13, 14]. TNF-*α* stimulates the expression of genes TNF-*α* involved in apoptosis and migration [11]. Therefore, inhibition of inflammatory factors can prevent the progression of DR [14]. Increased amounts of free oxygen radicals (FORs) lead to the development of DR and increased retinal damage [15]. Hyperglycemia also increases the amount of plasma-free radical and malondialdehyde (MDA) and disrupts the antioxidant system [12, 15]. Phytochemicals are compounds found in certain plants [16, 17]. Studies have shown that phytochemicals can prevent DR progression through their antiangiogenic, antioxidant, and anti-inflammatory characteristics [7, 18, 19]. In this paper, we intend to summarize an overview of the antiangiogenesis, anti-inflammatory, and antioxidant effects of phytochemicals on DR.

## 2. Antiangiogenic Phytochemicals against DR

Angiogenesis remains normal in the physiological state, but in disease conditions, the expression of proangiogenic factors such as VEGF, fibroblast growth factor (FGF), and others increases. Angiogenesis is often associated with malignancy and causes its progression [20, 21]. The formation of new vessels by germination is a process that has several stages, including the destruction of the basement membrane by enzymatic mechanisms, the proliferation of epithelial cells, migration, germination, the formation of branches and tubes [22]. Aloe-emodin is a derivative of anthraquinone and is found in various plants, including *Aloe vera*. Recent studies suggest that aloe-emodin is effective in treating many diseases, including cancer, viral, inflammatory, bacterial, parasitic, neurological, and hepatic diseases [23]. Aloe-emodin significantly reduced VEGF secretion, hypoxia-inducible factor- (HIF-) 1*α*, and *prolyl hydroxylase* domain protein 2 (PHD-2) expression in cultured ARPE-19 cells under hypoxia condition, thus reducing retinal angiogenesis. In a rat model of oxygen-induced retinopathy (OIR), oral intake of aloe-emodin reduced retinal neovascularization [24].

Andrographolide, a diterpenoid lactone, was obtained from *Andrographis paniculata*. It is effective in treating many diseases, including cancer, rheumatoid arthritis (RA), and upper respiratory tract infections [25]. Administrating andrographolide to STZ-induced DR mice inhibited retinal angiogenesis and vessel leakage. Andrographolide could also decrease VEGF and tissue factor (TF) expression, which played an important role in the process of angiogenesis [26]. Biochanin is an isoflavonoid obtained from *Trifolium pratense* and has various effects such as cancer prevention, nervous system protection, estrogen-like activity, anti-inflammatory, and antidiabetic [27]. In STZ-induced DR rats, oral administration of biochanin suppressed retinal TNF-*α* and IL-1*β* levels. Also, in the rats treated with this compound, VEGF levels and angiogenesis rate reduced, which led to improvements in symptoms in the retina [2]. *Vaccinium virgatum* contains high levels of anthocyanins, and their use has many effects on human health, including anticancer, neuroprotective, antioxidant, and blood pressure-lowering [16, 28]. In high glucose-induced HRCECs, the use of blueberry anthocyanin extract (BAE) and malvidin-3-glucoside (Mv-3-glc) controlled angiogenesis by reducing the content of VEGF and protein kinase B-1 (Akt) [16]. Chebulagic acid (CA), chebulinic acid (CI), and gallic acid (GA) are the active compounds in the alcoholic extract of Triphala, which suppresses the transmission of epithelial cells to the mesenchymal cells in retinal pigment cells [29]. In RF/6A cells under TNF-*α*, induced conditions intake of these three phytochemicals reduced matrix metalloprotease-9 (MMP-9) expression and angiogenic activity. Also, CA and CI reduced the expression of proangiogenic growth factor (PDGF-BB). These phytochemicals exerted their antiangiogenic effects by inhibiting the phosphorylation of p38, ERK, and NF-*κ*B [30]. Chlorogenic acid is a polyphenolic compound found in coffee, and its consumption is associated with a reduced risk of cardiovascular disease and type 2 diabetes. Also, it is used to treat obesity in the daily diet [31, 32]. The use of chlorogenic acid inhibited the proliferative capacity, cell migration, and tube formation induced by VEGF in HRECs and monkey choroid-retinal endothelial cell line (RF/6A). It also reduced the amount of phosphorylated VEGF receptor 2 (VEFGR2), mitogen-activated extracellular regulated kinase (MEK1/2), ERK1/2, p38, the activity of microglia cells, and the expression of VEGF in these cells. Similarly, in STZ-induced DR C57BL/6 mice, it was demonstrated that, by receiving chlorogenic acid orally, retinal neovascularization decreased [5]. Chrysin is a flavonoid found in many natural products, including honey and many plants [33]. So far, many benefits have been discovered for this phytochemical, including anticancer, antioxidant, antiapoptotic, and treatment of movement problems [34, 35]. In glucose-exposed RPE cells, treatment with chrysin reduced the production of VEGF and insulin-like growth factor-I (IGF-1). In diabetic mice, treatment with chrysin improved the reduced thickness of the retina and also increased the secretion of pigment epithelium-derived factor (PEDF), which is a neovascular suppression factor. The expressions of various enzymes involved in the visual cycle, such as RPE65, level of lecithin retinol acyltransferase (LRAT), and retinol dehydrogenase 5 (RDH5), were increased due to chrysin administration and led to improved vision in diabetic mice. Chrysin also reduced advanced glycation end (AGE) secretion, AGE receptor (RAGE) induction, and endoplasmic reticulum (ER) stress caused by increased glucose in human retinal pigment epithelial (HRPE) cells and improved the visual cycle [3]. In another model of DR in vitro, the use of chrysin in HRMVECs in hyperglycemic conditions inhibited the apoptosis of retinal endothelial cells. It also decreased the expression of HIF-1*α* and VEGF and the Ang-Tie-2 pathway. Oral administration of chrysin in db/db mice reduced the expression of VEGFR-2, HIF-1*α*, and VEGF and increased the expression of intercellular-binding proteins, including VE-cadherin and ZO-1 junction proteins and platelet endothelial cell adhesion molecule-1 (PECAM-1), and thus, it was effective in inhibiting vascular leakage. The amount of cellular adhesion proteins reduced the loss of pericytes and endothelial cells and ultimately inhibited the formation of acellular capillaries [36]. Curcumin is a polyphenolic compound obtained from *Curcuma longa*. This phytochemical has a wide range of effects, including anti-inflammatory, antimicrobial, and antioxidant used in the treatment of cancer [37]. Oral administration of curcumin reduced retinal capillary BM thickness and alleviated angiogenesis in the retina of diabetic rats by decreasing VEGF expression [7]. Decursin is a pyranocoumarin obtained from the roots of *Angelica gigas* [38]. Decursin was demonstrated to reduce the expression of vascular endothelial growth factor receptor (VEGFR-2) and tube formation in human retinal microvascular endothelial cells (HRMVECs) in vitro and Sprague–Dawley (SD) rats in vivo [39]. Formononetin is an isoflavones compound *Astragalus membranaceus* and is found in various plants. It is widely used in traditional medicine, has antioxidant, anticarcinogenic, and neuroprotective effects, and is used in the treatment of neurodegenerative diseases such as Alzheimer's [40, 41]. In cultured ARPE-19 cells under hypoxia conditions, the use of formononetin reduced the expression of HIF-1*α* and inhibited VEGF secretion. In addition, intraperitoneal injection of formononetin reduced neovascularization in OIR model rats [41]. Genistein is an isoflavone mainly derived from *Glycine max*. It has many biological benefits, including anticancer activity [42]. Genistein affects acute retinal pigment epithelial-19 cells (ARPE-19), cells treated with normal and high glucose were evaluated, and the results showed a significant reduction in VEGF levels and retinal angiogenesis [42]. Gentiopicroside (GP) is extracted from the root of Gentianaceae and has antioxidant and liver protection effects. The main ingredient in this substance is iridoid glycosides [43]. In diabetic rats, oral use of GP decreased VEGF levels and significantly improved PEDF expression [13]. Hesperidin and its aglycone hesperetin are two citrus flavonoids, which have biological effects against many diseases such as cardiovascular diseases and cancer [44]. Findings showed that hesperidin (100 and 200 mg/kg/day) could normalize VEGF level and retinal and plasma abnormalities in streptozotocin- (STZ-) induced DR rats through downregulation of retinal angiogenesis. Blood retinal barrier permeability and retinal thickness were also increased after hesperidin treatment [45]. Hesperetin administration to STZ-induced DR rats led to inhibition of retinal angiogenesis, dilation of the vessels, and thickening of the basement membrane (BM). VEGF and protein kinase C *β* (PKC*β*) were also inhibited by hesperetin which are the basic mechanisms for angiogenesis [46]. Kaempferol is a flavonoid compound found in many plants, including cabbage, beans, cumin, and onions. Kaempferol has various pharmacological effects, including anticancer, antimicrobial, antioxidant, anti-inflammatory, and protection of vital organs, including skin, liver, colon, ovary, pancreas, stomach, and bladder [47, 48]. Therefore, in many cancers, it is recommended to eat foods rich in kaempferol [48]. Kaempferol treatment inhibited the ability of cellular reproduction and migration due to increased glucose in HREC cells. The findings also suggested that kaempferol inhibited angiogenesis in HREC cells under high glucose conditions by suppressing VEGF and placenta growth factor (PGF) expression. High glucose could increase the activation of extracellular regulated protein kinase (Erk1/2), Src, protein kinase B-1 (Akt1), and phosphoinositide 3-kinases (PI3K) expression, and kaempferol could reverse these effects [1]. Scutellarin is a flavonoid glucuronide that has several pharmacological activities, including antioxidant, anti-inflammation, vascular relaxation, and antiplatelet [49]. Scutellarin administration to human retinal endothelial cells (HRECs) downregulated VEGF and hypoxia-inducible factor-1*α* (HIF-1*α*). As a result, scutellarin showed an inhibitory effect on retinal angiogenesis, proliferation, and tube formation [50]. In HRC cells under hypoxia and hyperglycemia condition, treatment with scutellarin reduced cell proliferation and migration, tube formation, and VEGF expression, thereby exerting its antiangiogenic effect. This compound also inhibited phosphorylation of extracellular regulated protein kinase (ERK), p-Src, and focal adhesion kinase (FAK) in these cells [9].

## 3. Antioxidant Phytochemicals against DR

The hyperglycemic conditions in diabetes lead to disruption of the electron transport chain and ultimately abnormal function of the retinal mitochondria, which in turn increases reactive oxygen species (ROS) production. Inflammatory pathways, oxidative stress, and ER stress regulate the level of autophagy and in severe stress conditions cause cell death and severe tissue damage [51]. Alpha-mangostin (*α*-MG) is a xanthoid that is isolated from *Garcinia mangostana* and has anti-inflammatory, antioxidant, and anticancer effects [52]. In STZ-induced DR rats, treatment with alpha-mangostin increased antioxidant capacity by reducing MDA level [53]. In a study, it was demonstrated that the main constituents of blueberry anthocyanins such as BAE, Mv, Mv-3-glc, and malvidin-3-galactoside (Mv-3-gal) significantly reduced ROS and by facilitating the expression of CAT and SOD antioxidant enzymes minimized the damaging effects of oxidative stress on HRCECs. Malvidin (Mv) decreased the production of superoxide products by reducing NADPH oxidase 4 (Nox4), but Mv-3-glc did not affect the Nox4 inhibiting. Mv, Mv-3-glc, and Mv-3-gal could also significantly alleviate nitric acid levels [16]. Curcumin use in STZ-induced DR rats increased antioxidant capacity and GSH levels in the retina. It also improved the elevated levels of nitrotyrosine and 8-OHdG [54, 55]. Curcumin significantly improved retinal dilatation and other vascular disorders caused by diabetes. Antioxidant enzymes that were reduced during diabetes were also compensated. Increased inflammatory factors such as TNF-*α*, VEGF, and BM thickness were also decreased after the use of curcumin. Curcumin administration also improved vascular endothelium and cytoplasmic damage in pericytes shown by retinal microscopic observations [54]. In STZ-induced PDR Wistar rats, oral administration also played a vital role in reducing oxidative stress by reducing MDA and increasing SOD, total antioxidant capacity (T-AOC) activity, and the ratio of B-cell lymphoma 2 (Bcl-2) to BCL-associated X (Bax) [7]. GSH, SOD, and CAT activation were also induced by curcumin [54]. In HG-induced ARPE-19 cells, curcumin treatment significantly reduced ROS production [12]. Consumption of curcumin in alloxan-induced DR rats increased the number of retinal ganglion cells under oxidative stress by increasing Brn3a expression [56]. Docosahexanoic acid (DHA) is a polyunsaturated fatty acid that is synthesized from the plant linolenic acid's fatty acid and is essential for the growth and development of the brain and retina that is also beneficial for pregnancy and lactation [57, 58]. *β*, *ε*-Carotene-3,3′-diol is a carotenoid with anti-inflammatory activity, with the ability to improve and prevent diseases that affect eye health and vision [59]. Studies on the effects of DHA and *β*, *ε*-carotene-3,3′-diol in STZ-induced male Wistar rats showed that these compounds exerted their antioxidant effects by weakly producing MDA and increasing GPx and GSH expression. In addition, *β*, *ε*-carotene-3,3′-diol could reduce the concentration of nitrotyrosine, while DHA had no such effect. They also improved the morphological changes caused by diabetes in the retina [60]. Eriodictyol is a flavonoid present in many citrus vegetables and medicinal plants and has anti-inflammatory, anticancer, antioxidant, and antiobesity effects [61]. The use of eriodictyol in rat retinal ganglion cells (RGCs) in hyperglycemic conditions increased the activity of antioxidant markers such as SOD, glutathione peroxidase (GPx), and CAT and ultimately reduced ROS production. Eriodictyol exerted its antioxidant activity by activating the Nrf2/HO-1 pathway [62]. Gastrodin is a phenolic glycoside, extracted from *Gastrodia elata*. The anti-inflammatory, antioxidant, and apoptotic effects of this compound have been identified. This phytochemical reduced apoptosis in HG-induced HRECs by regulating the sirtuin 1 (SIRT1)/Toll-like receptor 4 (TLR4)/NF-*κ*Bp65 pathway, increasing Bcl-2/Bax, and decreasing cytochrome C and cleaved caspase-3 expression. Administration of gastrodin also increased antioxidant capacity by reducing ROS production and decreased gene expression involved in oxidative stress such as HO-1, NQO1, and *γ*-glutamate-cysteine ligase modifier (GCLM) [63]. Genistein could inhibit the increased level of aldose reductase (ALR) caused by hyperglycemic conditions in ARPE-19 cells [42]. Genistein combined polysaccharide (GCP) is a drug rich in *Glycine max* isoflavones and has three main isoflavones, including genistein, daidzein, and glycetein. The use of this drug in the treatment of prostate cancer facilitates cell apoptosis and inhibits cell proliferation [64]. GCP caused an enhancement in antioxidant enzymes such as GSH-Px in postmenopausal women with DR [65]. The use of GP in STZ-induced DR rats was associated with low ROS production by regulating the levels of oxidative stress markers such as GSH, SOD, CAT, MDA, and carbonyl protein [13]. Administration of hesperidin in STZ-induced rats increased antioxidant capacity by decreasing aldose reductase (AR) activity and MDA as well as increasing superoxide dismutase (SOD) expression [45]. Hesperetin treatment in STZ-induced DR rats minimized cellular damage by affecting the activity of antioxidant enzymes such as total glutathione (tGSH), superoxide dismutase (SOD), and catalase (CAT) [66]. Lithospermic acid B (LSB) is a polyphenolic compound extracted from *Salvia miltiorrhiza* and was used in ancient China to treat heart diseases [67, 68]. Lithospermic acid administration in Otsuka Long-Evans Tokushima Fatty (OLETF) rats, an animal model of type 2 diabetes, was associated with a decrease in 8-hydroxy-2′-deoxyguanosine (8-OHdG), a marker of oxidative stress [69]. Naringin is a bioflavonoid used in the treatment of age-related diseases and has anticancer, anti-inflammatory, antiapoptotic, antiulcer, and antiosteoporotic effects [70, 71]. In STZ-induced rats, as well as in retinal Müller cells under hyperglycemic conditions, treatment with naringin activated antioxidant enzymes such as SOD and GSH [10]. Physcion 8-O-*β*-glucopyranoside (PG) is an anthraquinone, with a wide range of effects including anti-inflammatory, antioxidant, neuroprotective, and anticancer, causing liver and kidney toxicity and genetic damage [72]. Administration of PG on APRE-19 cells exposed to high glucose inhibited oxidative stress disorders in cells by reducing ROS [73]. Pterostilbene is a polyphenolic compound that, due to its two methoxyl groups, has broad effects in the prevention of type 1 and type 2 diabetes and hepatic steatosis and also reduces low-density lipoproteins (LDL) serum level [74]. In HRECs in hyperglycemic conditions, treatment with pterostilbene reduced ROS production by facilitating SOD activation [75]. Puerarin is an isoflavonoid derived from the root of *Pueraria montana* var*. lobata* and has various effects, such as antiaging, antidiabetic, and antihepatotoxic. This compound also inhibits cell proliferation by regulating mitogen-activated protein kinase (MAPK), mTOR/p70S6K, and Erk1/2 pathways and is used in cancer treatment [76]. Retinal pericyte cells exposure to AGE-BSA resulted in phosphorylation and activation of NADPH oxidase subunits, including p47phox and Rac1, so the production of ROS and the activity of enzymes involved in this pathway were stimulated. Administration of puerarin in these cells inhibited the activation of the NF-*κ*B pathway, phosphorylation of p47phox, and Rac1 and ultimately reduced ROS production. In rats' eyes injected with AGE-RSA, puerarin also reduced the apoptosis of pericyte cells and decreased the expression of 8-OHdG [77]. Quercetin is a polyphenolic flavonoid with heart-protective, antioxidant, anti-inflammatory, antimicrobial, and antidiabetic effects [78–81]. The use of quercetin by regulating miR-29b, PTEN/AKT, and NF-*κ*B pathways reduced the amount of ROS in ARPE-19 cells that were in hyperglycemic conditions [82]. Sauchinone is an anthocyanin extracted from *Saururus chinensis* and has been used in the past to treat fever, jaundice, and inflammatory diseases. This phytochemical is also useful for the treatment of hepatocellular carcinoma [83, 84]. Treatment of HG-induced ARPE-19 cells with sauchinone improved viability, reduced ROS production that facilitates the activity of antioxidant enzymes such as SOD, GPx, and CAT, and activated the Akt/Nrf2/HO-1 pathway. The rate of apoptosis in ARPE-19 cells is also alleviated by decreasing Bcl-2 and increasing Bax [85]. Scutellarin reduced the amount of ROS that induced the production of HIF-1*α*. It also inhibited oxidative stress-induced damage in HRECs under high glucose and hypoxic condition by reducing nicotinamide adenine dinucleotide phosphate (NADPH) oxidase activity [50]. Sinomenine is an isoquinoline and one of the main components of *Sinomenium acutum*, widely used in the treatment of rheumatism. This substance also has anti-inflammatory, analgesic, antiangiogenic, and immunosuppressive effects [86, 87]. Treatment with sinomenine in retinal microglia cells in hyperglycemic conditions reduces the production of ROS and limits the damage caused by oxidative stress [88]. Sulforaphane is an isothiocyanate found in broccoli sprouts and is involved in preventing tumor growth, reducing inflammation, and oxidative stress [89, 90]. The use of this compound in STZ-induced DR SD rats and high glucose-induced retinal Müller cells enhanced antioxidant capacity by improving the binding of nuclear erythroid type 2 factor (Nrf2) to antioxidant response elements (AREs) and transcription of the heme-oxygenase-1 (HO-1) and NADPH quinone oxidoreductase 1 (NQO1) genes. Another effect of sulforaphane in diabetic rats was to facilitate the activation of antioxidant enzymes such as GSH, SOD, and CAT [91]. Taxifolin is a flavonoid compound present in olive oil and onion with a wide range of effects in improving inflammation, cancer, and diabetes [92]. In diabetic rats with alloxan, the use of taxifolin reduced MDA levels and increased tGSH level. Thus, oxidative stress was greatly alleviated [15].

## 4. Anti-Inflammatory Phytochemicals against DR

Activation of Müller glial cells, which produces inflammatory cytokines, triggers the inflammatory response in diabetic retinopathy. And the accumulation of these cytokines increases the apoptosis of neurons in the retina [93]. Increased levels of ICAM-1 produced by retinal endothelial cells and leukostasis in the retinal capillaries trigger inflammation [94]. Administration of *α*-MG via gavage in STZ-treated rats had anti-inflammatory and antiglycation effects, which were manifested by a decrease in AGE, RAGE, TNF-*α*, and VEGF [53]. Diabetic retinopathy increases nuclear factor *κ*B (NF-*κ*Bp65), an inhibitor of kappa B (I*κ*B), and I*κ*B kinase (I*κ*K) expression in mice. However, the use of andrographolide in these models negatively regulates the NF-*κ*Bp65, I*κ*B, and I*κ*K expression. The findings suggested that increased levels of IL-1*β*, IL-6, and TNF-*α* in mice with DR were significantly reduced by intraperitoneal injection of andrographolide, and therefore, this phytochemical reduced retinal inflammation. Early growth response-1 (Egr1) expression, which regulated the production of inflammatory cytokines, also decreased by andrographolide [26]. Baicalein is a flavone derived from the root of the plant *Scutellaria baicalensis* and has antiviral, antioxidant, and anticancer effects, which is also used in the treatment of cerebral ischemia due to its neuroprotective effect [95]. Treatment with baicalein in STZ-induced DR rats improved morphological changes, including decreasing the thickness of the inner and outer layers of the photoreceptors and the number of retinal ganglion cells and increasing the presence of pyknotic nuclei. This combination also improved vascular abnormalities. It was also involved in reducing the proliferation and activation of microglial cells. Baicalein downregulated glial fibrillary acidic protein (GFAP), VEGF, IL-18, TNF-*α*, and IL-1*β*, thereby reducing the inflammatory response [96]. In STZ-induced DR in rats, oral administration of biochanin suppressed retinal TNF-*α* and IL-1*β* levels, so this phytochemical improved the complications caused by inflammatory processes [2]. Mv and Mv-3-glc showed their anti-inflammatory effects by suppressing ICAM-1 expression and activating NF- *κ*B in HRCECs treated with high glucose [16]. Treatment with chebulagic acid (CA), chebulinic acid (CI), and gallic acid (GA) in TNF-*α*-exposed RF/6A cells reduced the expression of inflammatory cytokines such as IL-6, IL-8, and monocyte chemoattractant protein-1 (MCP-1) by reducing phosphorylation of the p38, ERK, and NF-*κ*B. In addition, CA and CI also reduced the number of inflammatory factors such as eotaxin, macrophage inflammatory protein-1 (MIP-1b), and RANTES and increased anti-inflammatory factors such as IL-10 and IL-13 [30]. Curcumin consumption in STZ-induced DR rats reduced vascular leakage. NF-*κ*B is a factor that causes the secretion of proinflammatory cytokines, such as VEGF, inducible nitric oxide synthase (iNOS), and intercellular adhesion molecule-1(ICAM-1). Calcium/calmodulin-dependent protein kinase II (CaMKII) was also a factor that promoted retinal vascular damage. High levels of glucose increased the phosphorylation of these two factors. Treatment with curcumin in STZ-induced DR rats and Müller retinal cells in hyperglycemic conditions inhibited NF-*κ*B phosphorylation and ultimately reduced inflammatory factors, such as VEGF, iNOS, and ICAM-1 [97]. The use of curcumin in alloxan-induced diabetic rats improved disorders of retinal morphology. It also reduced leakage in retinal vessels. Curcumin increased the number of ganglion cells under oxidative stress by increasing Brn3a expression. It exerted its anti-inflammatory effects by reducing VEGF, iNOS, and ICAM-1. It also elevated RecA expression and vascular density. The use of curcumin in cultured RGC cells derived from the retinas of diabetic rats increased neurite outgrowth in these cells [56]. ARPE-19 cell exposure to high glucose enhanced ROS/PI3K/AKT/mTOR signaling pathway and as a result increased TNF-*α*, IL-6, and IL-1*β* [12]. Treatment with curcumin in STZ-induced DR Lewis rats showed that it can inhibit the production of inflammatory cytokines, including IL-1*β* and NF-*κ*B, and decrease VEGF expression [55]. Curcumolide, a sesquiterpene, extracted from *Curcuma wenyujin* has a wide range of effects, including anticancer, anti-inflammatory, and antioxidant [97]. The use of curcumolide in STZ-induced DR rats resulted in decreased expression of ICAM-1, leukocyte counts, and ultimately attenuated retinal vascular leakage [98]. In TNF-*α*-stimulated human umbilical vein endothelial cells (HUVECs), the use of this phytochemical inhibited the activity of p38 MAPK and NF-*κ*B and proinflammatory factors [14]. As previously has been described, eriodictyol blocked apoptosis and has antioxidant properties. In line with this data, eriodictyol played an important role in the alleviation of the inflammatory response in HG-induced RGCs by reducing the production of inflammatory cytokines, including TNF-*α* and IL-8. The anti-inflammatory and antiapoptosis effects were facilitated by activation of the Nrf2/HO-1 pathway by this drug [62]. In STZ-induced DR rats treated with genistein, inhibition of TNF-*α* expression and release, as well as decreased retinal microglial cell activation, was observed. The use of genistein in cultured microglial cells treated with glycated albumin also reduced TNF-*α* release by inhibiting the phosphorylation of ERK and P38 MAPKs [99]. Gentiopicroside (GP) significantly limited inflammatory responses by attenuating the expression of NF-*κ*B, TNF-*α*, IL-1*β*, and ICAM-1. In diabetic rats, oral use of GP decreased VEGF levels and significantly improved PEDF expression. The use of GP in the culture of retinal Müller cells under hyperglycemic conditions was associated with improved cell survival [13]. ICAM-1, TNF-*α*, and IL-1*β* were increased in the retina of diabetic rats. Administration of hesperetin reduced the levels of TNF-*α* and IL-1*β* and expression of caspase-3 in retinal Müller and astrocyte cells. This phytochemical also downregulated GFAP, AQP4, and capillary BM thickness and finally improved the morphological changes in the retinal layers [45, 66]. The use of lithospermic acid in OLETF rats caused morphological changes in the retina, including decreased vascular leakage, capillary atherosclerosis, and VEGF expression and increased thickness of the nerve layer, ganglion cells, and capillary BM layer. This phytochemical was derived from *Salvia miltiorrhiza* and reduced the serum levels of inflammatory markers, including urinary 8-OHdG, high-sensitivity C-reactive protein (hsCRP), MCP1, and TNF-*α* [69]. The use of *β*, *ε*-carotene-3, 3′-diol in STZ-induced DR rats reduced the amount of nitrotyrosine, which indicated the progression of inflammation [60]. Intraperitoneal injection of naringin in STZ rats increased ganglion cell count and ganglion cell layer (GCL) thickness and decreased GFAP levels. This compound inhibited the elevated level of retinal NF-*κ*B p65 caused by diabetes. In rat Müller cell line (rMC1) exposed to diabetic conditions, the use of naringin significantly inhibited inflammation by reducing inflammatory cytokines, such as TNF-*α*, IL-1*β*, and IL-6. It also reduced NF-*κ*B p65 transmission from the cytoplasm to the nucleus. In STZ-induced DR rats, treatment with naringin decreased the number of inflammatory cytokines mentioned above [10]. Paeoniflorin is a monoterpene glycoside phytochemical that is isolated from *Paeonia lactiflora* and has anti-inflammatory effects. It is also helpful in diseases that affect the immune system, such as RA and systemic lupus erythematosus (SLE) [100, 101]. Paeoniflorin inhibited MMP-9 activation in BV2 cells and the transmission of NF-*κ*B p65 to the nucleus and decreased p-p38 expression. It also increased the expression of suppressor of cytokine signaling 3 (SOCS3), an intracellular inhibitor of cytokines, by activating the TLR4 receptor. Other effects of paeoniflorin in STZ mice included reduced IL-1*β* and IBA-1 levels in inflammation and activation of microglial cells. This phytochemical also activated MMP-9 and increased SOCS3 expression in diabetic mice. Changes in the retina of diabetic mice, which included increased retinal thickness and retinal gliocyte proliferation, were also ameliorated by paeoniflorin [102]. The use of PG in APRE-19 cells treated with high glucose regulates the noncoding RNA activated by DNA damage (NORAD)/miR-125/STAT3 pathway. TNF-*α* and IL-1*β* levels and cellular apoptosis were also downregulated after treatment with PG [73]. The use of pterostilbene in HRECs under high glucose conditions reduced the proliferation of these cells, the production of TNF-*α* and IL-1*β*, and ultimately the inflammatory response. The reduction of NF-*κ*B transcription was another benefit of this compound [75]. The effects of quercetin on miRNAs in different inflammatory conditions were previously studied [80]; miRNAs have vital regulatory roles in different diseases and conditions [103]. Treating APRE-19 cells in hyperglycemic conditions with quercetin led to the suppression of apoptosis and proinflammatory cytokines such as MCP-1 and IL-6, as well as inhibition of ROS production while upregulating miR-29b expression. Moreover, miR-29b overexpression resulted in the promotion of the PTEN/AKT pathway and inhibition of the NF-*κ*B pathway in quercetin-treated cells. Taken together, quercetin exerted protective effects on DR via regulating the miR-29b, PTEN/AKT, and NF-*κ*B pathways [82]. Resveratrol is a phytoestrogen that has anti-inflammatory, antioxidant, and neuroprotective effects and can regulate brain activity due to its ability to cross the blood-brain barrier [104]. Administration of resveratrol in STZ-induced DR rats had a wide range of effects, including decreased expression and activation of NF-*κ*B, levels of inflammatory mediators such as TNF-*α*, IL-6, and prostaglandin-endoperoxide synthase 2 (COX-2), and mitigation of retinal cell apoptosis [19]. Sesamin, a lignan, is the main component of *Sesamum indicum*, has neuroprotective, antioxidant, and anti-inflammatory effects, and has been used in the treatment of patients with osteoarthritis in the past [105, 106]. Intraperitoneal injection of sesamin into STZ-induced DR mice reduced the activation of retinal microglia cells and proinflammatory factors such as TNF-*α* and ICAM-1. The antioxidant effect of this drug was manifested by decreasing the expression of inducible nitric oxide synthase (iNOS) and finally reducing the amount of ROS [107]. Shikonin, a naphthoquinone, is extracted from the root of *Lithospermum erythrorhizon* and has anti-inflammatory and anticancer effects. It has been used in the past to treat dermatitis, skin wounds, and burns [108]. Administration of shikonin in STZ mice could control the increased permeability of blood vessels in the retina. Diabetes reduced the thickness of the inner and middle layers of the retina, and the use of shikonin improved these effects. Oral use of shikonin reduced the expression of inflammatory factors such as COX-2, iNOS, and proapoptotic protein Bax, which were increased due to diabetes in the retina. It also reduced edema and damage to retinal cells. Shikonin treatment of RF cells in hypoxia and high glucose conditions reduced cell damage and also alleviated the expression of inflammatory factors such as COX-2, iNOS, and myeloperoxidase (MPO), and ZO-1, a protein for strong intercellular binding, is reduced in diabetic condition, while shikonin prevented changes in ZO-1 level [8]. Silybin is also known as silibinin, is a flavonolignan, and has been widely used to treat nonalcoholic fatty liver. Consumption of this phytochemical, obtained from *Silybum marianum*, also has anticancer and antidiabetic effects [109, 110]. The use of silybin in the treatment of STZ-induced DR rats reduced the number of obliterated retinal capillaries and improved leukostasis in retinal vessels. Administration of this compound also suppressed inflammation in the retina by reducing ICAM-1 expression [111]. Microglial cell culture in hyperglycemic conditions increased the expression and regeneration of inflammatory cytokines, including TNF-*α*, IL-1*β*, and IL-6. Treatment with sinomenine reduced the levels of cytokines. Sinomenine also attenuated NF-*κ*B p65 nuclear transport [88]. Administration of sulforaphane in STZ-induced DR SD rats significantly increased the number of ganglion cells. The anti-inflammatory effects of this compound could be concluded by observing the effects of sulforaphane on the reduction of TNF-*α*, IL-6, and IL-1*β*. In diabetic rats, the expression of four inflammatory factors, including pyrin domain-containing 3 (NLRP3), adaptor protein apoptosis-associated speck-like protein (ASC), cleaved caspase-1 p20 level, and IL-1*β* p17 increased. The use of this phytochemical in Müller cells in hyperglycemic conditions reduced the inflammatory response by lowering TNF-*α* and IL-6 levels [91]. Taxifolin improved inflammatory responses by significantly reducing IL-1*β* and TNF-*α*. Tissue damage to the retina also progressed to normalization with taxifolin. Congested and dilated arteries in the ganglion cell layer of the retina were also greatly improved after treatment with taxifolin in alloxan-induced DR rats [15]. Zerumbone, a monocyclic sesquiterpene, has been used in the past to treat diseases such as stomach pain, diabetes, and skin diseases. It has anti-inflammatory, antimicrobial, and antihypersensitivity effects [112]. Consumption of oral zerumbone extracted from *Zingiber zerumbet* in STZ-diabetic rats improved atrophy in the layers of nerve fibers and ganglion cells and increased retinal thickness. Decreased expressions of RAGE, TNF-*α*, IL-1*β*, IL-6, VEGF, ICAM-1, and vascular cell adhesion molecule-1 (VCAM-1) were other effects of this drug. Also, a decrease in NF-*κ*B apoptotic activity after using this compound could be seen in diabetic rats [113].

## 5. Conclusion

Diabetic retinopathy is one of the most important complications in patients with diabetes and can greatly affect a person's quality of life. Current therapeutic techniques for diabetic retinopathy are anti-VEGF injection, photocoagulation, and administration of corticosteroids, which have been demonstrated to be effective; however, they possess significant limitations and serious side effects such as invasiveness, short-lasting mechanism, retinal detachment, blurred vision, fungal infection burning the retina, and loss of vision [114–116].

**Figure 1:**
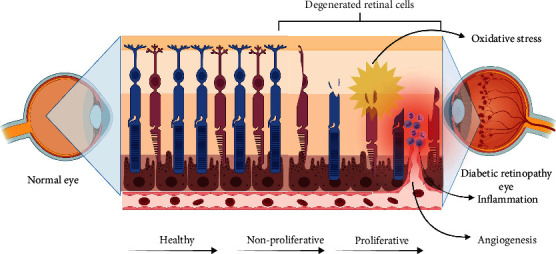
An insight into the molecular mechanism of angiogenesis, inflammation, and oxidative stress in the pathophysiology of diabetic retinopathy.

**Figure 2:**
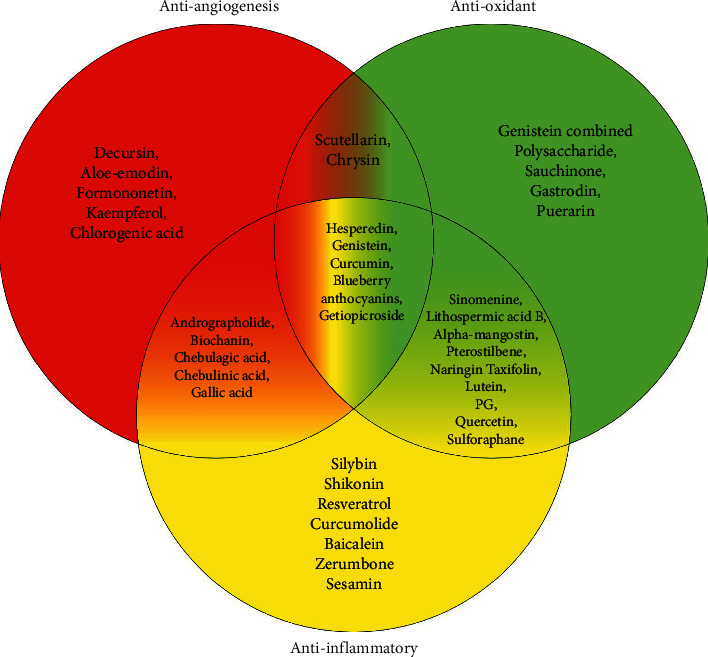
Simultaneous and individual effects of phytochemicals on angiogenesis, inflammation, and oxidative stress in diabetic retinopathy.

The present review provides an overview of the anti-inflammatory, antiangiogenesis, and antioxidant effects of phytochemicals (Table 1) in DR *in vitro* and *in vivo*. In Table 2, the effects of different phytochemicals on DR pathogenesis were summarized. Different types of phytochemicals including flavonoids, polyphenols, lignan, pyranocoumarin, iridoid glycosides, xanthoid, phytoestrogen, anthraquinone, naphthoquinone, sesquiterpene, monoterpene glycoside, isothiocyanate, anthocyanins, and isoquinoline inhibited angiogenic factors, such as VEGF, PKC*β*, and HIF-1*α*, as well as oxidative stress by affecting the production of ROS and activity of antioxidant enzymes, including SOD, MDA, NADPH oxidase, and CAT. Furthermore, the inflammatory factors, including TNF-*α*, IL-6, and IL-1*β*, which could damage the retina, were also downregulated by phytochemicals. The molecular mechanisms of angiogenesis, inflammation, and oxidative stress in the pathophysiology of diabetic retinopathy are illustrated in Figure 1. Phytochemicals not only affect one of the pathogenic mechanisms of DR but also can affect several of them simultaneously (Figure 2).

Based on the existing literature about the simultaneous effects of phytochemicals on the pathophysiology of DR, the present review provides some suggestions about using phytochemicals with multiple targets as a supplementary therapy for DR. It is also recommended to use different phytochemicals to enhance synergy and cover all aspects of DR by targeting multiple targets to prevent the disease or shorten the recovery time.

Although phytochemicals have various benefits, many factors should be noticed regarding their safety and production quality. Therefore, further pharmacokinetic and pharmacodynamic studies on the use of these products are necessary. Meanwhile, further studies are needed to investigate their cellular and molecular targets, alone and simultaneously.

## Figures and Tables

**Table 1:** Chemical structures of different phytochemicals used for diabetic retinopathy treatment.

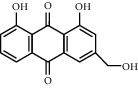	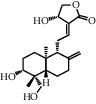	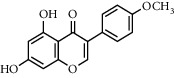	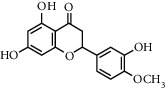	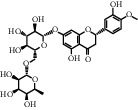
Aloe-emodin	Andrographolide	Biochanin	Hesperetin	Hesperidin
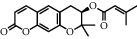	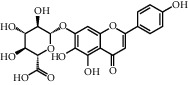	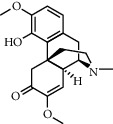	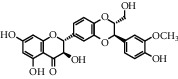	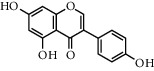
Decursin	Scutellarin	Sinomenine	Silybin	Genistein
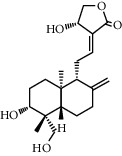	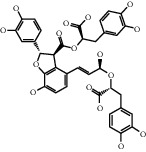	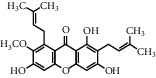	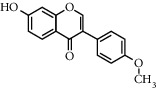	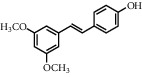
Andrographolide	Lithospermic acid B	Alpha-mangostin	Formononetin	Pterostilbene
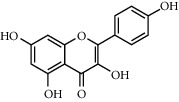	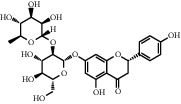	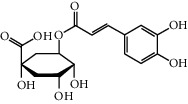	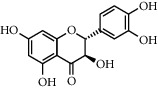	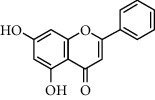
Kaempferol	Naringin	Chlorogenic acid	Taxifolin	Chrysin
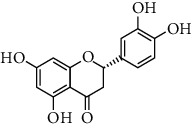	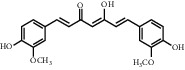	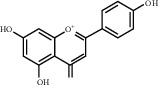	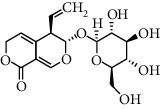	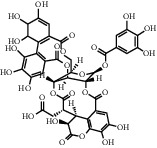
Eriodictyol	Curcumin	Anthocyanin	Gentiopicroside	Chebulagic acid
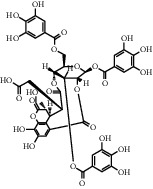	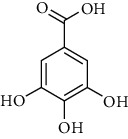	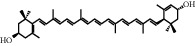	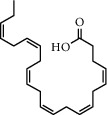	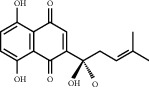
Chebulinic acid	Gallic acid	*β*, *ε*-Carotene-3,3′-diol	Docosahexaenoic acid	Shikonin
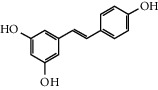	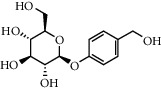	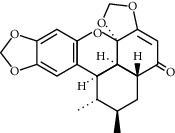	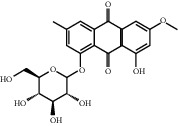	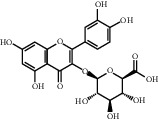
Resveratrol	Gastrodin	Sauchinone	Physcion 8-O-*β*-glucopyranoside	Quercetin
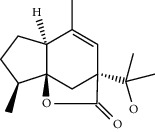	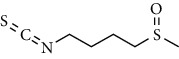	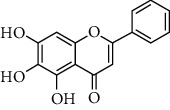	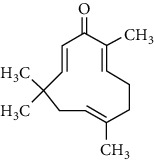	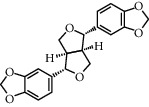
Curcumolide	Sulforaphane	Baicalein	Zerumbone	Sesamin

**Table 2:** Effects of different phytochemicals on diabetic retinopathy pathogenesis.

Phytochemical	Plant	Model	Dose/concentration	Study type	Mechanism	Reference
Aloe-emodin	—	ARPE-19 under hypoxia condition and OIR model rats	0.2–5.0 *µ*g/mL (in vitro) and 5.0 and 10.0 mg/kg/day (oral)	In vivo and in vitro	↓ Retinal neovascularization and expressions of VEGFA, HIF-1*α,* and PHD-2	Wu, 2016
Alpha-mangostin	*Garcinia mangostana*	STZ-induced DR SD rat	200 mg/kg/day	In vivo	↓ MDA, AGE, RAGE, TNF-*α*, and VEGF	Jariyapongskul, 2015
Andrographolide	*Andrographis paniculata*	STZ-induced NPDR and PDR C57BL/6 mice	10 mg/kg/day (intraperitoneally)	In vivo	↓ Retinal angiogenesis, inflammation, breakdown of BRB, VEGF, NF-*κ*B, Egr-1 phospho-NF-*κ*Bp65, IKK, TNF-*α*, IL-6, IL-1*β*, serpine1, and TF	Yu, 2015
Baicalein	—	STZ-induced DR SD female rats	150 mg/kg/d orally	In vivo	↓ Retinal inflammation, GFAP, VEGF, IL-18, TNF-a, IL-1, vascular abnormality, loss of ganglion cell layer (GCL), and microglial activation	Yang, 2009
Biochanin	—	STZ-induced DR Wistar rats	10 and 15 mg/kg/day (orally)	In vivo	↓ Retinal angiogenesis, inflammation, VEGF, TNF-*α*, and IL-1*β*	Mehrabadi, 2018
Blueberry anthocyanins	*Vaccinium virgatum*	High glucose-induced HRCECs	10 *μ*g/mL	In vitro	↓ Retinal inflammation, oxidative stress, ROS, and VEGF production ↑ CAT and SOD activity BAE, Mv, and Mv-3-gal ↓ Nox4 protein expression BAE, Mv-3-glc, and Mv-3-gal ↓ Akt expression, Mv Mv-3-glc, and Mv-3-gal ↓ ICAM-1 levels and NF-*κ*B	Huang, 2018
Chebulagic acid (CA), chebulinic acid (CI), and gallic acid (GA)	—	RF/6A cells were stimulated with TNF-*α* CAM model	1.0 to 100 *µ*M; 25, 25, and 100 *µ*M	In vivo and in vitro	↓ CA, CI, and GA: retinal angiogenesis, inflammation, expression of MMP-9, IL-6, IL8, MCP-1, phosphorylation of p38, ERK and NF-*κ*B CA, CI: eotaxin, MIP-1b, RANTES, PDGF-BB ↑ IL-10, and IL-13	Shanmuga, 2018
Chlorogenic acid	—	STZ-induced PDR C57BL/6 mice, HRECs, RF/6A cells, and microglia BV-2 cells	1,10 mg/kg/day (oral); 0.3125–0.5 *µ*M (in vitro)	In vivo and in vitro	↓ Retinal angiogenesis, microglia cell activation, VEGF expression, phosphorylation of VEGFR-2, MEK1/2, ERK1/2, and p38	Mei, 2018
Chrysin	—	HRMVEC and adult male db/db mice C57BLKS	1–20 *µ*M (in vitro); 10 mg/kg (oral)	In vitro and in vivo	↑ VE-cadherin, ZO-1 junction proteins, and PECAM-1 ↓ HIF-1*α*, VEGF, VEGF receptor-2, Ang-1, Ang-2, and Tie-2 proteins	Kang, 2016
Chrysin	—	HRPE cell db/db mice	1–20 *µ*M; 10 mg/kg/day (oral)	In vivo and in vitro	↓ Retinal neovascularization, VEGF, IGF-1, AGE secretion, RAGE, and ER stress ↑ Retinal thickness, PEDF, RPE65, LRAT, and RDH5 level	Kang, 2018
Curcumin	—	STZ-induced DR Lewis rats	0.5 g/kg, powdered diet	In vivo	↓ Retinal inflammation, 8-OHdG, nitrotyrosine, IL-1*β*, VEGF, NF-*κ*B, and ROS ↑ GSH activity	Kowluru, 2007
Curcumin	—	STZ-induced DR male Wistar albino rats	1g/kg body weight of rat orally	In vivo	↓ Retinal inflammation, HbA1c level, vessel diameter, thickening of BM, TNF-a, and VEGF ↑ GSH, SOD, and CAT activities	Suresh,2011
Curcumin	—	STZ-induced DR and rat retinal Müller cells	100 mg/kg/day (oral); 5–15 *μ*M	In vivo and in vitro	↓ Retinal vascular leakage, inflammation, VEGF, iNOS, ICAM-1expession, phosphorylation of CaMKII, and NF-*κ*B	Li, 2016
Curcumin	*Curcuma longa*	Alloxan-induced DR in rats and RGC from diabetic and normal rats	100 mg/kg/day (in vivo); 10–1000 nmol/mL (in vitro)	In vivo and in vitro	↓ Retinal inflammation, oxidative stress, retinal capillary basement membrane thickness, phosphorylation p65subunit of NF-*κ*B, CaMKII activity, VEGF, iNOS, and ICAM-1 expressions ↑ RecA and microvessel density, expression of Thy-1 and Brn3, and average neurite length	Pradhan, 2018
Curcumin	—	STZ-induced PDR Wistar rat	100 and 200 mg/kg/day (oral)	In vivo	↓ Retinal angiogenesis, oxidative stress, apoptosis, retinal capillary basement membrane thickness, VEGF, and MDA ↑ Ratio of Bcl-2 to Bax, SOD, and T-AOC	Yang, 2018
Curcumin	—	HG-induced ARPE-19 cells	0–20 *µ*mol/l	In vitro	↓ TNF-*α*, IL-6, IL-1*β*, and ROS-AKT/mTOR	Ran, 2019
Curcumolide	*Curcuma wenyujin*	STZ-induced DR, Wistar rats, and TNF-a-stimulated HUVECs	2.5–20 *μ*M intravitreal injection	In vivo and in vitro	↓ Retinal inflammation, leukostasis, vascular permeability, TNF-a, ICAM-1, p38 MAPK, and NF-*κ*B activation	Cai, 2017
Decursin	*Angelica gigas*	HRMVEC cells and STZ-induced SD rats	20 mg/kg/day (oral); 12.5–100 *µ*M (in vitro)	In vivo and in vitro	↓ Retinal proliferation and angiogenesis, VEGFR-2 expression, tube formation, and retinal neovascularization	Yang, 2013
(1) Docosahexaenoic acid (2) *β*, *ε*-Carotene-3,3′-diol	—	STZ-induced DR male Wistar rats	(1) CL and DL groups: 0.5 mg/kg (oral) (2) CDHA and DDHA groups: 13.3 mg/kg (oral)	In vivo	(1) ↓ MDA (1,2) and nitrotyrosine level (2) ↑ GPX activity and GSH level (1, 2)	Arnal, 2009
Eriodictyol	—	HG-induced rat RGCs	5–20 *μ*M	In vitro	↓ Retinal inflammation, production of ROS, TNF-*α*, IL-8, BAX, and cleaved caspase-3 ↑ SOD, GPX, CAT, Bcl-2, and activation of Nrf2/HO-1	Lv, 2019
Formononetin	*Astragalus membranaceus*	ARPE-19 cells under chemical hypoxia and OIR model rats	5.0 and 10.0 mg/kg/day (intraperitoneal); 0.2–5 *μ*g/mL (in vitro)	In vivo and in vitro	↓ Retinal neovascularization, VEGF, HIF-1*α*, and PHD-2	Wu, 2016
Gastrodin	*Gastrodia elata*	HG-induced HRECs	0.1–10 and 100 *µ*M	In vitro	↓ TLR4/NF-*κ*B p65 signaling pathway, ROS, NADPH, NQO1, Nrf2, and GCLM ↑ SIRT1	Zhang, 2018
Genistein	—	ARPE-19 cells treated with normal and high glucose concentrations	20 *µ*M	In vitro	↓ Retinal inflammation, angiogenesis, ALR, VEGF165, and VEGF secretion	Dongare, 2015
Genistein combined polysaccharides	—	12 postmenopausal Korean women	(Tablets) 2 g containing 120 mg of genistein and 57 mg of daidzein	In vivo	↑ SHBG and GSH-Px activity	Oh, 2005
Gentiopicroside	—	STZ-induced DR rats and rMC1 cells	20–80 mg/kg/day; 10–100 *µ*M (in vitro)	In vivo and in vitro	↓ Retinal inflammation, oxidative stress, overexpression of HDAC, ROS expression, MDA, protein carbonyl expression, NF-*κ*B, TNF-*α*, IL-1*β*, ICAM-1, GFAP, and VEGF expression ↑ HAT expression, GSH, SOD, CAT, and PEDF expression	Zhang, 2019
Hesperetin	—	STZ-induced DR Wistar rats	200 mg/kg/day (oral)	In vivo	↓ Retinal angiogenesis, dilated vessels, VEGF, PKC-*β*, vascular permeability, and thickening of BM	Kumar, 2012
Hesperetin	—	STZ-induced DR Wistar albino rats	100 mg/kg body (oral)	In vivo	↓ Retinal inflammation, ROS, TNF-*α*, IL-1*β*, caspase-3, GFAP, and AQP4 ↑ Retinal GSH SOD and CAT activity	Kumar, 2013
Hesperidin	—	STZ-induced DR SD rats	100 and 200 mg/kg/day (intragastrically)	In vivo	↓ Retinal angiogenesis, oxidative stress, inflammation, permeability of the BRB, VEGF, ICAM-1, TNF-*α*, IL-1*β*, AGE, AR activity, and MDA ↑ SOD and retinal thickness	Shi, 2012
Kaempferol	—	HRECs under high glucose condition	5–25 *µ*M	In vitro	↓ Retinal angiogenesis, proliferative ability, migration, VEGF and PGF expression, expression of PI3K, activation of Erk1/2, Src, and Akt1	Xu, 2017
Lithospermic acid B	*Salvia miltiorrhiza*	OLETF rats	10 or 20 mg/kg/day (oral)	In vivo	↑ Thickness of the nerve layer, ganglion cells, and capillary BM layer ↓ VEGF, hsCRP, MCP1, TNF-*α*, and 8-OHdG	Jin, 2014
Naringin	—	rMC1 STZ-induced DR rats	20–80 mg/kg/day (intraperitoneally); 50 *μ*M (in vitro)	In vivo and in vitro	↓ Retinal inflammation, oxidative stress, TNF-*α*, IL-1*β*, IL-6, NF-*κ*B level, and GFAP level ↑ SOD, GSH, GCL thickness, and ganglion cell number	Liu, 2017
Paeoniflorin	—	Microglia BV-2 cells and STZ-induced DR CD-1 mice	20 and 40 mg/kg/day (oral); 0.1–10 *μ*M	In vivo and in vitro	↓ Retinal inflammation, MMP-9 activation, expression of p-p38, NF-*κ*B translocation, IBA-1, and IL-1*β* ↑SOCS3 expression and activating TLR4	Zhu, 2017
Physcion 8-O-Β-glucopyranoside	—	HG-disposed APRE-19 cell injury	1.5 *µ*M	In vitro	↓ TNF-*α*, IL-1*β*, ROS generation, cell apoptosis, NORAD expression, and STAT3 ↑ miR-125 expression	Wan, 2020
Pterostilbene	—	HRECs under high glucose environment	1 mmol/l	In vitro	↓ TNF-*α*, IL-1*β*, ROS production, and NF-*κ*B mRNA and protein expression ↑ SOD activity	Shen, 2015
Puerarin	—	AGE-RSA induced bovine retinal pericyte cells AGE-RSA injected to rat eyes	1, 5, and 10 *µ*M; 400 mM (intravitreally)	In vivo	↓ NADPH oxidase activity, ROS, phosphorylation of p47 phox and Rac1, NF-*κ*B, and 8-OHdG	Kim, 2012
Quercetin	—	ARPE-19 cells were stimulated by high glucose	30 *μ*M	In vitro	↓ Cell apoptosis, MCP-1, IL-6, ROS, PTEN, p-p65, and I*κ*B*α* ↑ miR-29b expression and p-AKT	Wang, 2020
Resveratrol (trans-3,5,40-trihydroxystilbene)	—	STZ-induced DR rats	5 mg/kg per day orally	In vivo	↓ Retinal inflammation, NF-*κ*B, TNF-a, IL-6, and COX-2	Ghadiri Soufi, 2015
Sauchinone	—	Human RPE cell line/ARPE-19	5, 10, and 20 *µ*M	In vitro	↑ SOD, GPX, CAT, Bcl-2, P-Akt, nuclear nrf2, HO-1, and Akt/nrf2/HO-1 signaling pathway ↓ ROS and Bax	Shi, 2019
Scutellarin	*-*	HRECs under high glucose and hypoxic condition	0.1 nM; 1 *µ*M	In vitro	↓ Retinal angiogenesis and proliferation, tube formation, HIF-1*α*, VEGF, ROS, and NADPH oxidase activity	Wang, 2014
Scutellarin	—	HRECs under high glucose and hypoxic condition and STZ-induced DR	40 mg/kg/day (intragastrically); 10 *μ*M (in vitro)	In vivo and in vitro	↓ Retinal angiogenesis and proliferation, VEGF, phosphorylation of ERK, FAK, and Src	Long, 2019
Sesamin	—	STZ-induced DR mice	30 mg/kg BW (intraperitoneally), alternate day	In vivo	↓ Retinal inflammation, TNF-*α*, ICAM, microglia activation, and iNOS	Ahmad, 2016
Shikonin	—	STZ with whole-body hypoxia-induced DR in C57BL/6 mice and RPE cells	0.5–50 mg/kg/day (oral); 0.1–10 *μ*M (in vitro)	In vivo and in vitro	↓ Retinal inflammation, vascular permeability, cell loss, COX-2, iNOS, Bax, MPO, and ZO-1	Liao, 2017
Silybin	—	STZ-induced DR SD rats	15 and 30 mg/kg/day (orally)	In vivo	↓ Retinal inflammation, obliterated retinal capillaries, retinal vascular leukostasis, and ICAM-1	Zhang, 2014
Sinomenine	*Sinomenium acutum*	Retinal microglia cells isolated from SD rats	0.01–1 mM	In vitro	↓ Retinal inflammation, microglial activation, IL-1*β*, TNF-*α*, IL-6, and ROS	Wang, 2007
Sulforaphane	—	STZ-induced DR SD rats and rMC1	0.5 and 1 mg/kg/day 2.5 *μ*M (in vitro)	In vivo and in vitro	↓ Retinal inflammation, TNF-*α*, IL-6, IL-1*β*, NLRP3, ASC, and cleaved caspase-1 p20 level ↑Ganglion cells, antioxidant capacity, NRF2, transcriptions of HO-1, and NQO1	Li, 2019
Taxifolin	—	Alloxan-induced DR and albino Wistar male rats	50 mg/kg/day (oral)	In vivo	↓ Retinal inflammation, MDA, IL-1*β,* and TNF-*α* ↑ tGSH level	Ahiskali, 2019
Zerumbone	*Zingiber zerumbet*	STZ-induced DR and male Wistar rats	40 mg/kg once a day orally	In vivo	↓ Retinal inflammation, NF-*κ*B, apoptosis, thickness of retinal layers, AGEs, TNF-*α*, IL-1*β*, IL-6, VEGF, NF-*κ*B, HbA1c, RAGE, ICAM-1, and VCAM-1 protein expression	Tzeng, 2016

^*∗*^↓: decrease; ↑: increase.

## Data Availability

No data were used to support this study.
